# Tetrasodium EDTA central venous catheter lock solution in home parenteral nutrition patients: Ease of use and patient satisfaction, a prospective study

**DOI:** 10.1177/11297298251376970

**Published:** 2025-09-17

**Authors:** Lina Saucedo, Yasaman Ghorbani, Maria Heusser, Giulia Chagas, Celeste Arca, Katherine J P Schwenger, Johane P Allard

**Affiliations:** Department of Medicine, Division of Gastroenterology, Toronto General Hospital, University of Toronto, Toronto, ON, Canada

**Keywords:** Catheter lock solution, parenteral nutrition, acceptability, CRBSI

## Abstract

**Objective:**

Chronic intestinal failure is a devastating chronic medical condition, which requires central venous access for parenteral nutrition infusion. The presence of a central venous line is associated with an increased risk of bloodstream infection and thrombosis. Currently, the heparin lock is the most used catheter lock, but still has risks for line infection. The 4% tetrasodium EDTA catheter lock (KiteLock™) solution, has demonstrated a decrease in infection and thrombosis risk, but requires extra steps for the preparation and infusion. The objective of this study was to evaluate the patients ease-of-use and acceptability of the new KiteLock solution in the home parenteral nutrition (HPN) patients.

**Methods:**

This prospective, open-label, single-center study evaluated patient acceptability of tetrasodium EDTA (KiteLock) in HPN. Patients were contacted four times, beginning with an instructional visit by the HPN nurse. They then completed three 30-day phases: standard heparin/saline flush, tetrasodium with withdrawal (when feasible), and tetrasodium EDTA. During the final week of each phase, patients completed a phone survey and questionnaire with the HPN nurse.

**Results:**

Of the 21 patients enrolled, 14 completed the study. Seven did not: two withdrew due to time constraints, two found KiteLock difficult to infuse, and three had incomplete data. Among those who competed the study, some reported reduced ease-of-use with KiteLock (1 hard to open, 5 difficulties withdrawing, 1 overall difficulty), though most still rated it easy or very easy. Three patients continued to find it more difficult by study end. No adverse effects were reported. Preparation time decreased over time as patients adapted. Initial satisfaction with KiteLock was lower (50% find it neutral), but improved with familiarity, becoming comparable to heparin with 12 patients satisfied (*N* = 14).

**Conclusions:**

KiteLock solution is well accepted by the patient or caregiver, despite involving more steps to use it.

## Introduction

Chronic intestinal failure (CIF) is recognized as a rare disease and is a devastating chronic medical condition that occurs when the absorption of nutrients and fluids from the intestinal tract is inadequate to support hydration, growth, and survival.^1^ CIF can occur due to intestinal malfunction, such as malabsorptive conditions and dysmotility, or secondary to surgical resection.^1^ In both instances patients require prolonged parenteral nutrition (PN), which requires central venous access where a hyperosmolar nutrient solution can be infused.^2^ A variety of central venous catheters (CVC) can be used, including peripherally inserted central catheters (PICC), tunneled catheters, and implanted ports.^2^ However, the presence of CVC is associated with an increased risk of bloodstream infection and thrombosis,^3^ these complications occur frequently and can result in a cascade of other complications.^4^ The most common complication, and most frequent cause of hospital admission in home PN patients is catheter related bloodstream infection (CRBSI).^5^

To mitigate these risks, several strategies have been implemented. First, to prevent a line infection, a combination (i.e. bundle) of interventions (e.g. hand hygiene and sterile barrier precautions) are used for placement and aftercare of CVC to decrease the rates of CRBSI.^6^ The second strategy is properly choosing the device type (e.g. catheters with antibacterial or anti biofilm properties) and insertion site.^2^ For last, the catheter lock solution plays a role in the patency of the central line,^7^ mainly with activity against biofilms and anticoagulant properties, thus decreasing the rate of thrombosis and CRBSI.^8^ Indeed, the catheter lock therapy is recommended by the guidelines for the prevention of intravascular catheter related infections in the general catheter population^9^ and also in the home PN patients.^10^ An ideal catheter lock solution should have a large spectrum of activity against common pathogens, the ability to penetrate or disrupt biofilm, compatibility with anticoagulants, a prolonged stability, low risk of toxicity and adverse effects, a low potential for bacterial resistance and cost effectiveness.^8^ However, the ideal catheter lock solution is still a challenge to find among the general patient population using central catheter and more studies are needed.^8^

Currently, several new devices or solutions are being developed for use in preventing primary CRBSI, such as catheters with antibacterial and/or anti-biofilm properties and different catheter lock solutions.^11^ Antibiotic lock solutions have been used for salvaging CVCs in confirmed CRBSI.^8^ However, it is not recommended as a primary prophylaxis due to the increased risk of antibiotic-resistant organisms.^12^ According to the United States Center for Disease Control, antibiotics “should be used only to mitigate infection.”^13^ However, one antimicrobial solution, taurolidine, has been used successfully as a lock solution in preventing CRBSI without promoting emergence of resistant bacterial.^14^ Although an antibiotic may have anti-biofilm properties in high concentrations, it does not have anticoagulant properties, thus, giving protection against infection, but not against thrombosis.^15^ The use of 70% ethanol lock solutions has also been successful in reducing CRBSI, however, adverse events have been documented when used in polyurethane lines, and it doesn’t contain anticoagulant properties,^16^ leading to increased thrombosis risk.^17^ Conversely, the citrate and heparin lock solutions, which prevent line thrombosis through anticoagulant properties, lacks antimicrobial or anti-biofilm properties.^18^ Therefore, the need for a lock solution that both prevents line thrombosis and CRBSI is essential.^19^

Recently, a new catheter lock solution, the 4% tetrasodium EDTA catheter lock solution, KiteLock, has been approved by Canada Health for use in home PN patients.^20^ It has been tested in both adult and pediatric patients and has been shown to significantly decrease CRBSI and line thrombosis.^21,22^ KiteLock decreases biofilm formation as well as contains both antiseptic and anti-thrombotic properties.^23^ KiteLock is effective in preventing central catheter related infection because tetrasodium-EDTA highly chelates divalent cations. By binding intraluminal Ca²⁺, it blocks the Ca²⁺-dependent steps of the coagulation cascade, preventing fibrin-sheath and thrombus formation.^24^ Then, chelating the Ca²⁺/Mg²⁺ cross-bridges, which cement the extracellular polymeric matrix, collapses bacterial, fungal, and mixed-species biofilms, producing significant reductions in viable organisms on catheter surfaces.^23^ The same divalent-cation stripping property disrupts the lipopolysaccharide bridges which are required for stabilizing outer membrane of Gram-negative bacteria, leading to increased membrane permeability and microbial injury. Thus, KiteLock results in a broad non-antibiotic antisepsic.^25^ However, the KiteLock device is more difficult to manipulate.

For the regular heparin lock, the catheter needs to be with a pre-filled 10 mL sterile normal saline flush followed by 3–5 mL of pre-filled heparin syringe, whereas KiteLock requires extra preparation, per instructions from the manufacturer.^26^ This includes drawing up 3–5 mL of the Tetrasodium EDTA solution into an empty sterile syringe prior to flushing and locking the catheter. Prior to starting PN infusion, patient need to attach a new sterile empty syringe to the catheter, pull back the Tetrasodium EDTA solution from the catheter then, discard it.^26^ Catheter is flushed with 10 mL of sterile normal saline before starting the infusion.^26^ There are no known serious product-related adverse reactions when KiteLock is used as intended, however, paresthesia and/or dysgeusia has been described if the product unintentionally passes into the vein.^27^

Another important aspect to consider is that home PN patients differ from other patients with central lines at home, such as dialysis or chemotherapy patients. The self-care and autonomy on managing the central line is an important benchmark for the home PN patients. The patient’s independence can be defined by the Karnofsky score in a quality-of-life assessment.^28^ For most of the hemodialysis or chemotherapy patients, the procedures using a central line is done in a specialized center, with the assistance of specialized nurses.^9^ On the other hand, home PN patients can either administer the PN with the assistance of a nurse or by themselves and/or their primary caregiver, however the latter is more common.^28^ Therefore, patient and family education and competency are crucial.^29^ Since this is an independent procedure performed by the patient, the more complex preparation can lead to non-adherence to the treatment resulting in subsequent complications. Therefore, the objective of this study was to evaluate the patients’ ease-of-use and acceptability of the KiteLock solution and document any possible side effects compared to the heparin lock solution.

## Methods

### Study population

This is a prospective, open-label, single-center study of the patient acceptability of using tetrasodium EDTA catheter lock solution (KiteLock) in home PN patients that was conducted between February 2018 and September 2024 and received research ethic approval at University Health Network. All patients provided informed consent, and all procedures performed in studies involving human participants were in accordance with the ethical standards of the institutional and/or national research committee and with the 1975 Helsinki declaration and its later amendments or comparable ethical standards. The inclusion criteria for this study included adult patients ⩾18 years old who received HPN and were currently being treated with heparin lock and had been compliant with use for ⩾1 month before study entry. Exclusion criteria included use of other catheter locks, inability to give informed consent, alcohol or drug abuse, pregnant or lactating women, clinical instability, subjects who are hypersensitive or allergic to the product ingredients, active therapy with long-term anti-microbial, or actively participating in another HPN clinical trial which may interfere with results.

### Study design and data collection

This study was to investigate the use of tetrasodium EDTA catheter lock solution in home PN patients over a 3-month period. Consented patients began the study with 1 month (30 days) of using their usual standard-of-care heparin and saline flush to establish baseline characteristic, followed by 1 month (30 days) of tetrasodium solution with withdrawal (when possible), followed by 1 month (30 days) of tetrasodium EDTA solution. During the last week of each of the 3 months, the patients were asked to evaluate the lock and flush (see Supplemental Material Questionnaires 1–3). Patients were followed and treated according to usual care during the study, except for the catheter lock solution. This include following standard guidelines from ASPEN and ESPEN^30,31^ in monitoring and treating infectious and mechanical catheter-related complications as part of standard of care, with regular clinic visits and patient contact with the multidisciplinary team.

The study participants were contacted four times throughout the study. The first visit was conducted by the HPN nurse who would instruct the patients on the use of the new lock solution prior to starting the 3-month period. The following three phone calls were also conducted by the HPN nurse and occurred at the end of each month (30 days). At each phone visit the nurse recorded the time to perform the procedure and any symptoms or side effects that the patient had documented. In addition, the nurse administered a survey (see Supplemental Material Survey 1–3) and inquired about any interim event such as hospitalization, surgery, and/or illness (phone calls 2, 3, and 4). The catheter lock solution was distributed by Ontario Medical Supply with Home total PN solution and supplies, as per patient’s usual delivery schedule.

### Clinical outcomes

Regular home PN monitoring and bloodwork was drawn at least once at the beginning of the study and once at the end of the study (3 months later), or more frequently, as clinically indicated or as per the patient’s usual care. This monitoring includes an anthropometric assessment (weight, height, body mass index), diet recall, 24 h ostomy and urine output, and usual bloodwork with complete blood count, electrolytes, glucose, creatinine, and liver enzymes.

### Statistical analysis

Parameters of interest were compared between groups using Kruskal-Wallis test, Wilcoxon ranked sum, or χ^2^ and Fisher exact test, as necessary. Statistical significance was set at *p* < 0.05. Continuous variables were presented as median (1st quartile; 3rd quartile). Categorical variables were presented as *n* (percentage). Analysis of the data was performed in R 4.2.2.

## Results

A total of 21 patients were recruited during the study period and 14 patients completed the study. Out of the seven participants who did not complete the study, two patients withdrew their consent due to time constraints, two individuals found the KiteLock solution too difficult to infuse, and three patients had incomplete data. Baseline characteristics and the difference between those who completed the study versus those who dropped out can be found in Table 1 and Table S1. Overall, those who did not complete the study were significantly older and had significantly lower albumin and calcium. All other variables, including PN prescription, clinical and biochemical variables were comparable. The majority of patients received a 12-h overnight TPN infusion, typically administered from 8:00 p.m. to 8:00 a.m.

**Table 1.** Baseline demographic and clinical characteristics of patients who completed the study and those who did not.

Demographic and Clinical Characteristics	*n*	Overall (*N* = 21)	*n*	Completed (*N* = 14)	*n*	Incomplete (*N* = 7)	*p*-Value
Age (years)	21	46 (33, 62)	14	39 (31, 54)	7	65 (46, 70)	0.010
Female sex, *n* (%)	21	11 (52%)	14	8 (57%)	7	3 (43%)	0.659
Weight (kg)	20	61 (53, 72)	14	65 (50, 77)	6	60 (59, 71)	>0.999
Height (m)	21	1.65 (1.62, 1.72)	14	1.65 (1.60, 1.72)	7	1.65 (1.62, 1.72)	0.736
BMI (kg/m^2^)	20	22.9 (19.4, 25.2)	14	22.9 (18.9, 24.9)	6	22.9 (20.1, 25.5)	0.968
Charlson comorbidities index (points)	21	2 (0, 4)	14	2 (0, 3)	7	4 (2, 5)	0.068
*PN prescription*							
Years on PN (years)	21	2 (1, 3)	14	1.5 (1, 4)	7	2 (1, 3)	0.815
Days on PN (days)	21	6.00 (5.00, 7.00)	14	6.00 (4.00, 7.00)	7	7.00 (6.00, 7.00)	0.096
Days on extra hydration (days)	21	0.00 (0.00, 3.00)	14	0.50 (0.00, 4.00)	7	0.00 (0.00, 1.00)	0.407
kcal/day PN (kcal)	21	1,354 (963, 1,670)	14	1,087 (727, 1,421)	7	1,443 (1,162, 1,730)	0.110
kcal/kg/day (kcal)	21	22 (14, 28)	14	16 (13, 26)	7	23 (20, 29)	0.146
AA/day (g)	21	60 (46, 77)	14	58 (41, 75)	7	71 (50, 80)	0.232
AA/kg/day (g)	21	0.98 (0.76, 1.29)	14	0.97 (0.65, 1.30)	7	1.00 (0.98, 1.29)	0.332
Dextrose/day (g)	21	189 (129, 225)	14	147 (96, 223)	7	214 (180, 274)	0.101
Dextrose/kg/min (g)	21		14		7		0.145
Lipids/day (g)	21	40 (30, 45)	14	37 (24, 43)	7	43 (35, 51)	0.135
Lipids/kg/day (g)	21	0.60 (0.45, 0.75)	14	0.53 (0.35, 0.90)	7	0.72 (0.59, 0.75)	0.192
*Biochemical variables*							
Hemoglobin (g/L)	21	116 (97, 128)	14	120 (107, 128)	7	104 (92, 140)	0.550
Leucocytes (10^9^ cells/L)	21	6.10 (4.40, 7.00)	14	6.15 (4.40, 7.00)	7	5.20 (4.20, 6.50)	0.477
Platelets (10^9^ cells/L)	21	223 (171, 306)	14	232 (189, 306)	7	171 (124, 355)	0.400
Glucose (mMol/L)	20	5.30 (4.80, 5.70)	14	5.35 (5.10, 5.90)	6	4.50 (4.20, 5.50)	0.148
ALP (U/L)	21	110 (84, 153)	14	112 (84, 122)	7	107 (80, 167)	>0.999
GGT (U/L)	18	29 (20, 101)	12	27 (17, 80)	6	47 (24, 101)	0.482
ALT (U/L)	21	19 (14, 45)	14	24 (14, 52)	7	17 (10, 30)	0.332
AST (U/L)	10	18 (15, 33)	4	20 (15, 54)	6	18 (13, 33)	0.915
Bilirubin (µmol/L)	21	5.0 (3.0, 7.0)	14	6.0 (3.0, 10.0)	7	3.0 (3.0, 7.0)	0.170
Albumin (g/L)	21	41 (37, 42)	14	41 (40, 42)	7	37 (32, 39)	0.027
Sodium (mMol/L)	21	139 (137, 139)	14	139 (136, 139)	7	139 (137, 142)	0.339
Potassium (mMol/L)	21	4.50 (4.20, 4.60)	14	4.55 (4.20, 4.60)	7	4.30 (3.90, 4.50)	0.188
Chloride (mMol/L)	20	104.5 (101.0, 107.0)	14	104.0 (100.0, 107.0)	6	104.5 (104.0, 110.0)	0.340
CO_2_ (mMol/L)	19	23 (22, 26)	14	23 (22, 26)	5	23 (22, 26)	>0.999
Phosphate (mMol/L)	20	1.12 (1.03, 1.25)	14	1.12 (1.03, 1.33)	6	1.15 (1.03, 1.23)	0.869
Calcium (mMol/L)	20	2.25 (2.15, 2.33)	14	2.29 (2.21, 2.33)	6	2.11 (2.07, 2.25)	0.032
Magnesium (mMol/L)	21	0.89 (0.81, 0.97)	14	0.89 (0.81, 0.90)	7	0.97 (0.81, 1.02)	0.368
Creatinine (µmol/L)	20	85 (58, 99)	14	79 (60, 100)	6	85 (55, 97)	0.967
eGFR (mL/min)	20	83 (62, 112)	14	96 (69, 113)	6	63 (54, 68)	0.136
Urea (mMol/L)	7	6.80 (3.00, 7.40)	6	6.95 (3.00, 7.40)	1	5.20 (5.20, 5.20)	0.857
On antibiotics, *n* (%)	17	3 (18%)	13	3 (23%)	4	0 (0%)	0.541
Antibiotic type, *n*	3		3		0		n/a
Clavulin		2		2		0	
Metronidazole		1		1		0	
Reason for antibiotics, *n*	3		3		3		n/a
CRBSI		0		0		0	
Skin		1		1		0	
Small intestinal bacterial overgrowth		1		1		0	
Respiratory		1		1		0	
Catheter type, *n* (%)	21		14		7		0.574
PICC line		17 (81%)		12 (86%)		5 (71%)	
Hickman		4 (19%)		2 (14%)		2 (29%)	
Catheter localization, *n* (%)	21		14		7		0.830
Left arm		11 (52%)		8 (57%)		3 (42%)	
Right arm		6 (29%)		4 (29%)		2 (29%)	
Jugular		4 (19%)		2 (14%)		2 (29%)	
Number of lumens, *n* (%)	18		11		7		0.596
Single		5 (28%)		4 (36%)		1 (14%)	
Double		13 (72%)		7 (64%)		6 (86%)	
CRBSI last 6 months, *n* (%)	21	4 (19%)	14	4 (29%)	7	0 (0%)	0.255

Next, we wanted to investigate the clinical outcomes of those who completed the study. Overall, no serious adverse effects were recorded, and the most frequent new symptom reported during the KiteLock period was a metallic taste in the mouth. The metallic taste symptom was comparable to previous reports with the use of KiteLock solution.^21^ The outcomes between the heparin time period and the KiteLock time period were also comparable, see Table 2.

**Table 2.** Clinical parameters of those who completed the study.

Clinical Characteristics	*n*	Heparin/saline period	*n*	Kitelock + withdrawal period	*n*	Kitelock period
New symptoms, *n* (%)	14	0 (0%)	13	6 (46%)	11	4 (36%)
Symptom, *n* (%)						
Fever		0 (0%)		0 (0%)		
Pain		0 (0%)		0 (0%)		
Chills		0 (0%)		0 (0%)		
Metallic taste		0 (0%)		5 (35.7%)		
Lips tingling		0 (0%)		2 (14.3%)		
Cold		0 (0%)		1 (7.1%)		
Restless legs		0 (0%)		1 (7.1%)		
Admissions, *n* (%)	14	3 (21%)	14	0 (0%)	11	0 (0%)
Cause admission, *n* (%)	2		0		0	
GI surgery		0 (0%)		0 (0%)		0 (0%)
GI disease		0 (0%)		0 (0%)		0 (0%)
Infection		2 (100%)		0 (0%)		0 (0%)
Hepatobiliary		0 (0%)		0 (0%)		0 (0%)
Pulmonary		0 (0%)		0 (0%)		0 (0%)
Hematological		0 (0%)		0 (0%)		0 (0%)
Neurologic		0 (0%)		0 (0%)		0 (0%)
Cancer		0 (0%)		0 (0%)		0 (0%)
Other		0 (0%)		0 (0%)		0 (0%)
Thrombosis admission, *n* (%)	14	0 (0%)	14	1 (7.1%)	11	0 (0%)
New medication, *n* (%)	14	4 (29%)	14	3 (21%)	11	1 (9.1%)
New medication type, *n* (%)						
NSAID		0 (0%)		0 (0%)		0 (0%)
Opioids		0 (0%)		0 (0%)		0 (0%)
Antiemetics		0 (0%)		0 (0%)		
Antidepresives		0 (0%)		0 (0%)		
Antimotility		0 (0%)		0 (0%)		
Blood glucose		0 (0%)		0 (0%)		
CV agents		0 (0%)		0 (0%)		
Other GI agents		0 (0%)		0 (0%)		
Hormonals		0 (0%)		0 (0%)		
Vitamins		0 (0%)		0 (0%)		
Sleep		0 (0%)		0 (0%)		
Anticonvulsants		0 (0%)		0 (0%)		
Antibiotics		4 (100%)		2 (67%)		1 (100%)
Other		0 (0%)		1 (33%)		0 (0%)
New antibiotic, *n* (%)	14	4 (29%)	12	2 (17%)	11	2 (18%)
Antibiotic type, *n* (%)	4		2		2	
Ciprofloxacin		1 (25%)		0 (0%)		1 (50%)
Rifaximin		0 (0%)		1 (50%)		1 (50%)
Penicillin		3 (75%)		1 (50%)		0 (0%)
Reason for antibiotics, *n* (%)	4		2		2	
CRBSI		2 (50%)		0 (0%)		0 (0%)
UTI		0 (0%)		1 (50%)		1 (50%)
Skin		0 (0%)		0 (0%)		0 (0%)
SIBO		0 (0%)		1 (50%)		1 (50%)
Respiratory		2 (50%)		0 (0%)		0 (0%)
Other		0 (0%)		0 (0%)		0 (0%)
Venous line change, *n* (%)	14	3 (21%)	14	1 (7.1%)	11	1 (9.1%)
Reason for change, *n* (%)	3		1		1	
Infection		3 (100%)		0 (0%)		0 (0%)
Dysfunction		0 (0%)		0 (0%)		1 (100%)
Migration		0 (0%)		1 (100%)		0 (0%)
Other		0 (0%)		0 (0%)		0 (0%)

**Figure 1.**
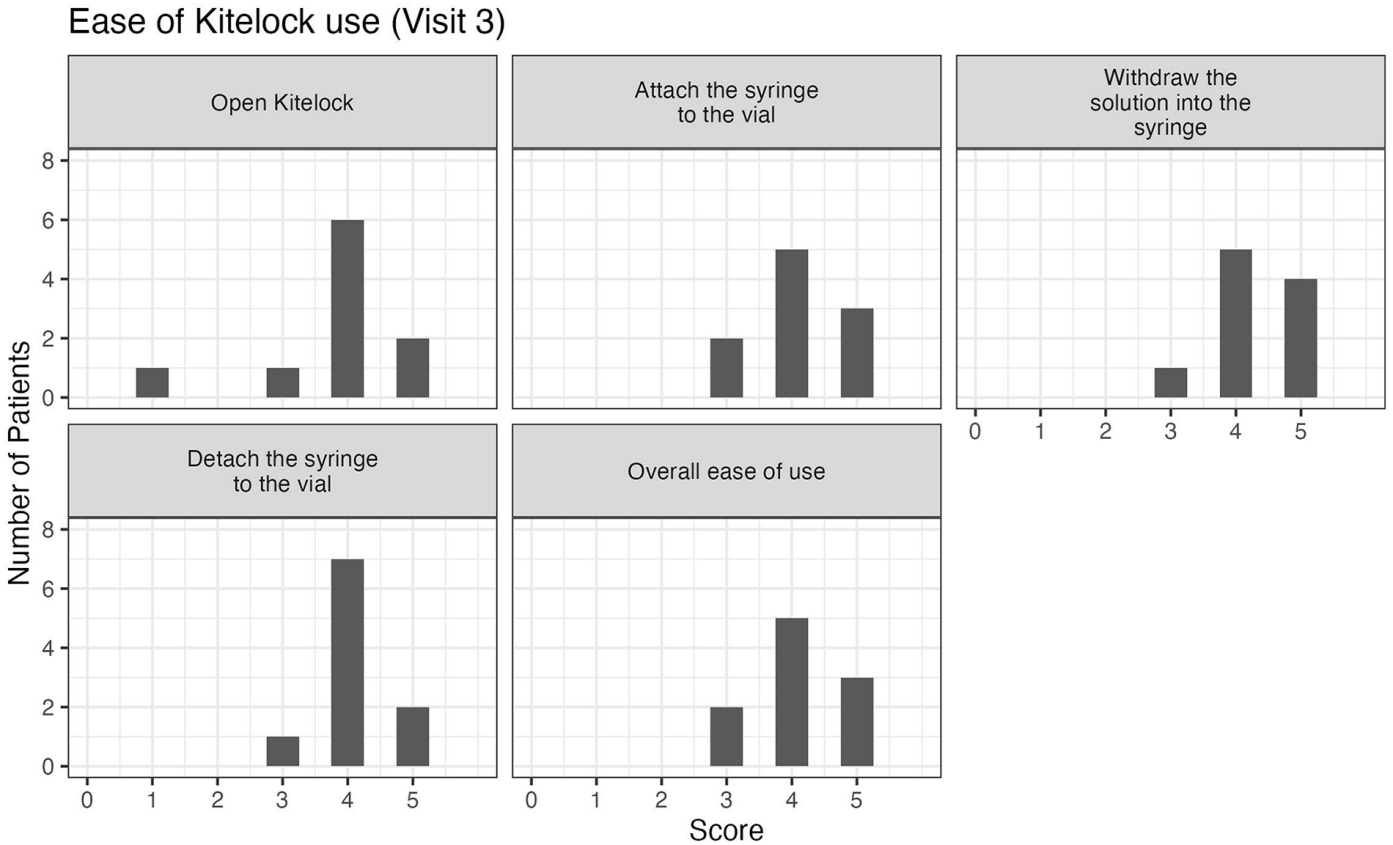
Ease of KiteLock use during the first months of switching from Heparin to KiteLock (Visit 3). The ease of use was scored on a scale of 1–5, with 1 being classified as the least easy to use. *N* = 14. 1 = Very hard, 2 = Hard, 3 = Not hard, not easy, 4 = Easy, 5 = Very easy.

**Figure 2.**
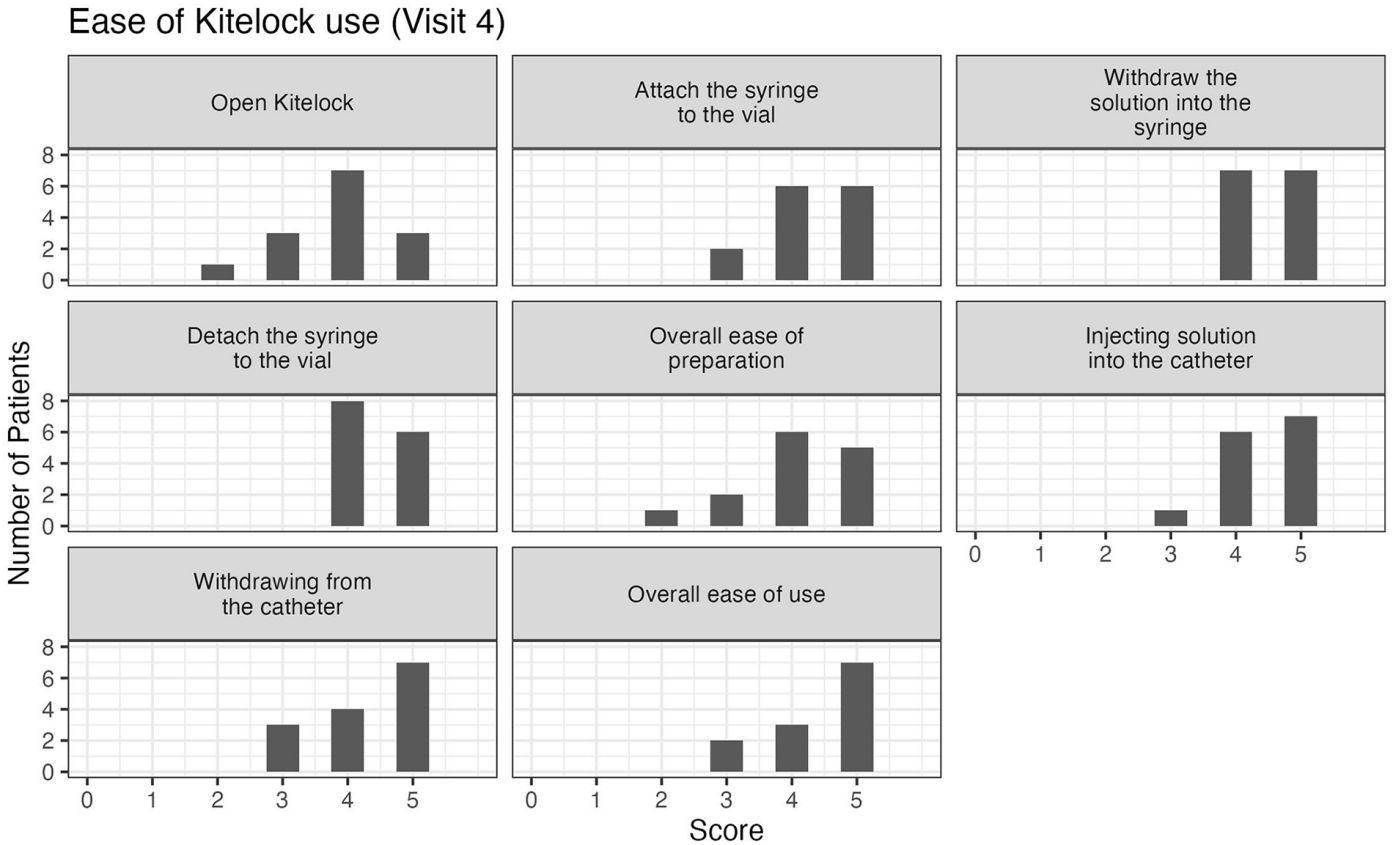
Ease of KiteLock use by the end of last month of the study (Visit 4). The ease of use was scored on a scale of 1–5 with 1 being classified as the least easy to use. *N* = 14. 1 = Very hard, 2 = Hard, 3 = Not hard, not easy, 4 = Easy, 5 = Very easy.

We also wanted to investigate the ease-of-use. When asking patients about the traditional heparin lock, all patients ranked this lock as either easy or very easy to use for: preparing the heparin syringe and attaching the syringe to the catheter; flushing the heparin into the catheter; detaching the syringe from the catheter and for the overall use (see Figure S1). When the heparin catheter lock was switched to the KiteLock, the ease-of-use decreased. Specifically, one patient found the KiteLock solution container hard to open, five patients found withdrawing the KiteLock solution from the catheter hard to do, and one patient found the overall ease-of-use hard. Nonetheless, most patients still found KiteLock easy or very easy to use (see Figure 1). By the final month of the study, three participants still found KiteLock more difficult to use (see Figure 2). However, when comparing the time to administer the lock, connect and disconnect the lock there was no significant difference between heparin lock and KiteLock (see Table 3). Moreover, as we recorded the time to administer the lock at the beginning and at the end of the month, we noted the times for the procedure decrease during the month, being comparable to the previous time on heparin lock.

**Table 3.** Times to administer lock solution.

Times to Administer Lock Solution	*n*	Heparin/saline	*n*	KiteLock + withdrawal	*n*	KiteLock	*p*-Value
Time to connect FM, seg (s)	13	300 (210, 480)	13	330 (180, 600)	13	180 (94, 331)	0.409
Time to disconnect FM, seg (s)	11	180 (120, 308)	11	320 (117, 600)	11	121 (60, 600)	0.690
Time to connect LM, seg (s)	13	300 (205, 480)	13	240 (190, 600)	11	169 (60, 420)	0.292
Time to disconnect LM, seg (s)	11	165 (120, 300)	11	240 (115, 600)	11	120 (60, 212)	0.369

FM: first Monday of the month; LM: last Monday of the month.

**Figure 3.**
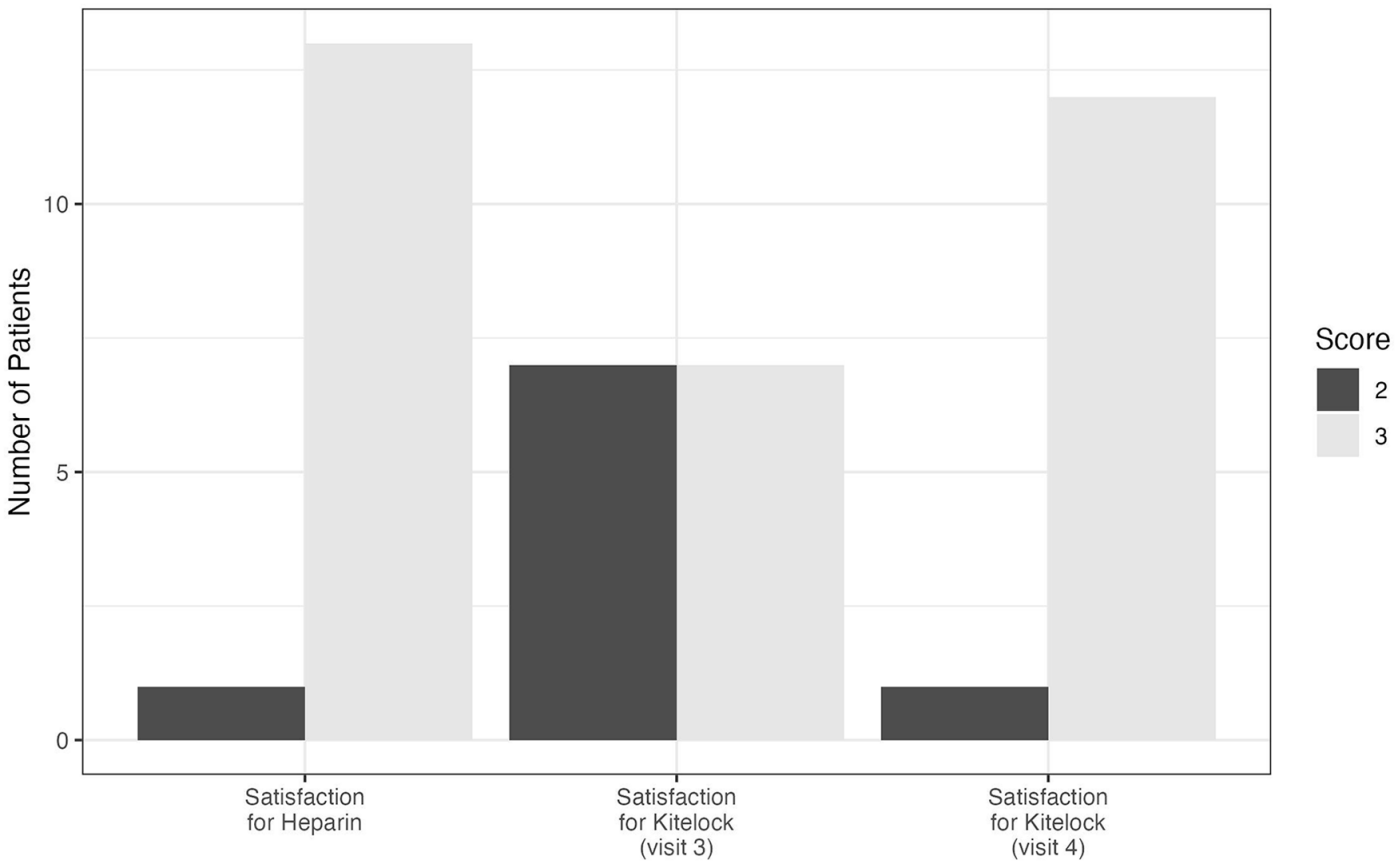
Patient satisfaction for heparin and KiteLock. The patient satisfaction was scored on a scale of 1–3 with 1 being the lowest satisfaction. *N* = 14. 1 = Very hard, 2 = Hard, 3 = Not hard, not easy, 4 = Easy, 5 = Very easy.

Finally, we wanted to understand the overall satisfaction of using KiteLock versus heparin. We found that all but one individual were satisfied with heparin. When KiteLock was first initiated, satisfaction dropped, with only 50% being satisfied. However, as individuals became accustomed to the new lock, the overall satisfaction became comparable to heparin (see Figure 3).

## Discussion

Overall, we found that in those who completed the study, the Kitelock solution was well accepted by the patient or caregiver, despite its increased complexity of use. Patients who did not complete the study were found to be significantly older, to have a significantly lower serum albumin and calcium, and a trend toward a higher Charlson comorbidity index.

The patients in this study were comparable to the general home PN population.^28,32^ In our study we found that the median age was 46 years and 52% were female, which is similar to other published studies.^28,32^ Regarding home PN indication, the most frequent cause for home PN was short bowel syndrome^28,32–34^ and the venous access was PICC and tunneled catheters.^28,32^ When comparing the PN prescription, our findings were very similar to the Canadian PN prescription in previous registry,^28^ but slightly different than the US registry^32^ where total PN calories and proteins were slightly higher; 22 versus 33 kcal/kg^32^ and protein 94–108 versus 66 g. This could reflect the country preferences for specific PN prescriptions, or the fact that the mean body mass index for the US registry was higher than in our registry, 24 kg/m^2^,^32^ versus 22 kg/m^2^. In similar literature there was no information focused on the catheter lock solution used.

When comparing the patients who completed the study to those who dropped out, we found that those who dropped out were significantly older, had significantly lower serum albumin and calcium, and had a higher, but not significantly, Charlson comorbidity index. This could be explained by the fact that as patients age, their medical condition becomes more complex,^29^ which can lead to an increased burden of care,^29^ as reflected by the increased Charlson comorbidity index. Furthermore, the significantly lower albumin and calcium further highlights increased medical complexity^35,36^ and nutritional status. Therefore, when considering whether to change a patient’s device/solution, the care team should consider to weigh the risks and benefits for the individual, especially if they are older and more complex medically.

In this observational study, we wanted to assess the ease of use of the KiteLock solution as a lock for the venous catheters in the home PN patients, compared to the previous experience using heparin lock. As the PN patients are more independent and self-sufficient to manage their infusions and lock solutions, the introduction of a new lock solution (KiteLock) could be challenging to use regardless of previous clinical trials demonstrating a decrease in the incidences of CRBSI and line thrombosis.^21,22^

In those who completed the study, the general ease of use was comparable between the KiteLock and heparin lock, after the 3-month period. However, when the KiteLock solution was first introduced, study participants noted that it was less easy than the heparin lock, indicating that there is a learning curve associated with switching lock solutions. Specifically, as the patients increase their use of the KiteLock solution, the ease of use increased with infusing it and the time to connect and disconnect decreased. Overall, by the end of the study period, the overall satisfaction was comparable between KiteLock and heparin lock solutions.

To our knowledge, there are no studies looking at KiteLock acceptance in parenteral nutrition patients. In a pediatric study with dialysis therapy, there was an increase in catheter lock solution viscosity during cap changes after KiteLock solution introduction, but there was no difficulty aspirating the solution from the catheter and no significant adverse events or changes in hemodialysis treatment parameters.^37^ In that study, considering these issues, acceptance of the KiteLock solution was not assessed.

Although the study objective was to evaluate ease of use, we also measured the effectiveness of KiteLock against CRBSI. Similar to other studies,^22,38^ we found that individuals had a lower rate of infections on KiteLock, although this did not reach statistical significance due to the small sample size. In these studies, the rates of infection reported were 14 CLABSI in the pre-intervention period, to 3 in the first 12 month post-intervention period (*p* = 0.04) compared to 2 CLABSI in the heparin/saline period to 0 in the KiteLock withdrawal period and KiteLock period (*p* non statistical significance).^22,38^

Our study is not without limitations. This was a single-center observational study with a small sample size, describing patient experience. There was not enough power to assess differences in rate of CRBSI. Home PN patients is a small population and intestinal failure requiring PN is considered a rare disease. In addition, patients have complex medical issues, all of which makes recruitment challenging. However, the ease-of use has never been evaluated before and shows different aspects of home catheter management, focusing on patient perspective which is not frequently assessed. Future studies should include multiple centers and a larger sample size.

Overall, this study suggests that the KiteLock solution is well accepted by the patient or caregiver, despite the more step-requiring instructions to use. This is in addition to the known benefits of KiteLock solution which is mainly the prevention of CRBSI and line thrombosis.^20,22,37,38^ Future studies should include multiple sites and a larger sample size.

## Supplemental Material

sj-pdf-1-jva-10.1177_11297298251376970 – Supplemental material for Tetrasodium EDTA central venous catheter lock solution in home parenteral nutrition patients: Ease of use and patient satisfaction, a prospective study Supplemental material, sj-pdf-1-jva-10.1177_11297298251376970 for Tetrasodium EDTA central venous catheter lock solution in home parenteral nutrition patients: Ease of use and patient satisfaction, a prospective study by Lina Saucedo, Yasaman Ghorbani, Maria Heusser, Giulia Chagas, Celeste Arca, Katherine J P Schwenger and Johane P Allard in The Journal of Vascular Access

## References

[1] Pironi L Arends J Bozzetti F , et al. ESPEN guidelines on chronic intestinal failure in adults. Clin Nutr 2016; 35: 247–307. 26944585 10.1016/j.clnu.2016.01.020

[2] Pittiruti M Hamilton H Biffi R , et al. ESPEN guidelines on parenteral nutrition: central venous catheters (access, care, diagnosis and therapy of complications). Clin Nutr 2009; 28: 365–377. 19464090 10.1016/j.clnu.2009.03.015

[3] Dibb M Lal S. Home parenteral nutrition: vascular access and related complications. Nutr Clin Pract 2017; 32: 769–776. 29023196 10.1177/0884533617734788

[4] Brandt CF Tribler S Hvistendahl M , et al. Home parenteral nutrition in adult patients with chronic intestinal failure: catheter-related complications over 4 decades at the main Danish Tertiary Referral Center. JPEN J Parenter Enteral Nutr 2018; 42: 95–103. 29505150 10.1177/0148607116678766

[5] Ross VM Guenter P Corrigan ML , et al. Central venous catheter infections in home parenteral nutrition patients: outcomes from Sustain: American Society for Parenteral and Enteral Nutrition’s National Patient Registry for Nutrition Care. Am J Infect Control 2016; 44: 1462–1468. 27908433 10.1016/j.ajic.2016.06.028

[6] Selby LM Rupp ME Cawcutt KA. Prevention of central-line associated bloodstream infections: 2021 update. Infect Dis Clin North Am 2021; 35: 841–856. 34752222 10.1016/j.idc.2021.07.004

[7] Noelting J Jurewitsch B Allard JP. Non-antibiotic antimicrobial catheter lock solutions in patients on home parenteral nutrition. Nutrients 2018; 10: 1165. 30149607 10.3390/nu10091165 PMC6165181

[8] Justo JA Bookstaver PB. Antibiotic lock therapy: review of technique and logistical challenges. Infect Drug Resist 2014; 7: 343–363. 25548523 10.2147/IDR.S51388 PMC4271721

[9] O’Grady NP Alexander M Burns LA , et al. Guidelines for the prevention of intravascular catheter-related infections. Clin Infect Dis 2011; 52: e162–e193. 10.1093/cid/cir257 PMC3106269 21460264

[10] Daoud DC Wanten G Joly F. Antimicrobial locks in patients receiving home parenteral nutrition. Nutrients 2020; 12: 439. 32050544 10.3390/nu12020439 PMC7071146

[11] Wouters Y Causevic E Klek S , et al. Use of catheter lock solutions in patients receiving home parenteral nutrition: a systematic review and individual-patient data meta-analysis. JPEN J Parenter Enteral Nutr 2020; 44: 1198–1209. 31985068 10.1002/jpen.1761 PMC7540581

[12] Mermel LA Allon M Bouza E , et al. Clinical practice guidelines for the diagnosis and management of intravascular catheter-related infection: 2009 update by the Infectious Diseases Society of America. Clin Infect Dis 2009; 49: 1–45. 19489710 10.1086/599376 PMC4039170

[13] CDC. Antibiotic prescribing and use, www.cdc.gov/antibiotic-use/about/index.html (2024, accessed 31 May 2025).

[14] Liu Y Zhang AQ Cao L , et al. Taurolidine lock solutions for the prevention of catheter-related bloodstream infections: a systematic review and meta-analysis of randomized controlled trials. PLoS One 2013; 8: e79417. 10.1371/journal.pone.0079417 PMC3836857 24278133

[15] Droste JC Jeraj HA MacDonald A , et al. Stability and in vitro efficacy of antibiotic-heparin lock solutions potentially useful for treatment of central venous catheter-related sepsis. J Antimicrob Chemother 2003; 51: 849–855. 12654743 10.1093/jac/dkg179

[16] Mermel LA Alang N. Adverse effects associated with ethanol catheter lock solutions: a systematic review. J Antimicrob Chemother 2014; 69: 2611–2619. 24891431 10.1093/jac/dku182

[17] Sierra CM Rodriquez C Bahjri K. Ethanol lock for prevention of CVC-related bloodstream infection in pediatric patients: a systematic review and meta-analysis. J Pediatr Pharmacol Ther 2023; 28: 386–396. 38130502 10.5863/1551-6776-28.5.386 PMC10731934

[18] Zhao Y Li Z Zhang L , et al. Citrate versus heparin lock for hemodialysis catheters: a systematic review and meta-analysis of randomized controlled trials. Am J Kidney Dis 2014; 63: 479–490. 24125729 10.1053/j.ajkd.2013.08.016

[19] Niyyar VD Lok CE. Pros and cons of catheter lock solutions. Curr Opin Nephrol Hypertens 2013; 22: 669–674. 24100219 10.1097/MNH.0b013e328365ba53

[20] Ornowska M Wong H Ouyang Y , et al. Control of Line Complications with KiteLock (CLiCK) in the critical care unit: study protocol for a multi-center, cluster-randomized, double-blinded, crossover trial investigating the effect of a novel locking fluid on central line complications in the critical care population. Trials 2022; 23: 719. 36042488 10.1186/s13063-022-06671-5 PMC9425798

[21] Hill J Garner R. Efficacy of 4% tetrasodium ethylenediaminetetraacetic acid (T-EDTA) catheter lock solution in home parenteral nutrition patients: a quality improvement evaluation. J Vasc Access 2021; 22: 533–539. 32815457 10.1177/1129729820946916

[22] Hirsch TI Fligor SC Tsikis ST , et al. Administration of 4% tetrasodium EDTA lock solution and central venous catheter complications in high-risk pediatric patients with intestinal failure: a retrospective cohort study. JPEN J Parenter Enteral Nutr 2024; 48: 624–632. 38837803 10.1002/jpen.2644 PMC11216891

[23] Liu F Hansra S Crockford G , et al. Tetrasodium EDTA is effective at eradicating biofilms formed by clinically relevant microorganisms from patients’ central venous catheters. mSphere 2018; 3: e00525-18. 10.1128/mSphere.00525-18 PMC6262258 30487154

[24] Banfi G Salvagno GL Lippi G. The role of ethylenediamine tetraacetic acid (EDTA) as in vitro anticoagulant for diagnostic purposes. Clin Chem Lab Med 2007; 45: 565–576. 17484616 10.1515/CCLM.2007.110

[25] Shein AMS Wannigama DL Higgins PG , et al. Novel colistin-EDTA combination for successful eradication of colistin-resistant Klebsiella pneumoniae catheter-related biofilm infections. Sci Rep 2021; 11: 21676. 34737361 10.1038/s41598-021-01052-5 PMC8568960

[26] KiteLock4% Sterile Catheter Solution How to Use. https://sterilecareinc.com/wp-content/uploads/2020/08/WI-4-KiteLock-4-How-to-Use-HT01-27102016.pdf

[27] KiteLock4% Sterile Catheter Solution Evidence Summary. https://sterilecareinc.com/wp-content/uploads/2024/08/KiteLock-Evidence-Summary-v5.pdf

[28] Raman M Gramlich L Whittaker S , et al. Canadian home total parenteral nutrition registry: preliminary data on the patient population. Can J Gastroenterol 2007; 21: 643–648. 17948134 10.1155/2007/217897 PMC2658131

[29] Kumpf VJ Gray B Monczka J , et al. Parenteral nutrition at home/long-term parenteral nutrition. Am J Health Syst Pharm 2024; 81: S112–S120. 10.1093/ajhp/zxae081 PMC11170492 38527076

[30] Pironi L Boeykens K Bozzetti F , et al. ESPEN practical guideline: home parenteral nutrition. Clin Nutr 2023; 42: 411–430. 36796121 10.1016/j.clnu.2022.12.003

[31] Guenter P Worthington P Ayers P , et al. Standardized competencies for parenteral nutrition administration: the ASPEN model. Nutr Clin Pract 2018; 33: 295–304. 29570861 10.1002/ncp.10055

[32] Winkler MF DiMaria-Ghalili RA Guenter P , et al. Characteristics of a cohort of home parenteral nutrition patients at the time of enrollment in the sustain registry. JPEN J Parenter Enteral Nutr 2016; 40: 1140–1149. 25972431 10.1177/0148607115586575

[bibr33-11297298251376970] Bond A Soop M Taylor M , et al. Home parenteral nutrition and the older adult: experience from a national intestinal failure unit. Clin Nutr 2020; 39: 1418–1422. 31337513 10.1016/j.clnu.2019.06.019

[34] Bakker H Bozzetti F Staun M , et al. Home parenteral nutrition in adults: a European multicentre survey in 1997. ESPEN-Home Artificial Nutrition Working Group. Clin Nutr 1999; 18: 135–140. 10451476 10.1054/clnu.1999.0021

[35] Evans DC Corkins MR Malone A , et al. The use of visceral proteins as nutrition markers: an ASPEN position paper. Nutr Clin Pract 2021; 36: 22–28. 33125793 10.1002/ncp.10588

[36] Dalemo S Bostrom KB Hjerpe P. Plasma albumin and calcium concentrations, and long-term mortality in primary health care patients in Sweden. Scand J Prim Health Care 2020; 38: 430–438. 33226880 10.1080/02813432.2020.1843809 PMC7783069

[37] Robinson CH Harvey E Nemec R , et al. Use of 4% tetrasodium EDTA (KiteLock) to prevent central venous catheter-related bloodstream infections in pediatric hemodialysis patients. Pediatr Nephrol 2025; 40: 1081–1091. 39576326 10.1007/s00467-024-06601-4

[38] Quirt J Belza C Pai N , et al. Reduction of central line-associated bloodstream infections and line occlusions in pediatric intestinal failure patients receiving long-term parenteral nutrition using an alternative locking solution, 4% tetrasodium ethylenediaminetetraacetic acid. JPEN J Parenter Enteral Nutr 2021; 45: 1286–1292. 32770561 10.1002/jpen.1989

