# Supplementary material for: Tetrasodium EDTA central venous catheter lock solution in home parenteral nutrition patients: Ease of use and patient satisfaction, a prospective study
Source: J Vasc Access. 2025 Sep 17;27(3):939–48. doi: 10.1177/11297298251376970 (PMC13135650; doi:10.1177/11297298251376970)
Supplement: sj-pdf-1-jva-10.1177_11297298251376970 – Supplemental material for Tetrasodium EDTA central venous catheter lock solution in home parenteral nutrition patients: Ease of use and patient satisfaction, a prospective study [file sj-pdf-1-jva-10.1177_11297298251376970.pdf]

Table S1: Baseline clinical characteristics of patients who completed the study and those who did not

	n	Overall (N=21)	n	Completed (N=14)	n	Incomplete (N=7)	p-value
Comorbidities, n	21		14		7		n/a
Heart disease		7 (33%)		6 (43%)		1 (14%)	
Cerebral vascular accident		2 (10%)		2 (14%)		0 (0%)	
Dementia		0 (0%)		0 (0%)		0 (0%)	
Chronic obstructive pulmonary disease		2 (10%)		0 (0%)		2 (29%)	
Liver disease		2 (10%)		1 (7%)		1 (14%)	
Type 2 diabetes		2 (10%)		1 (7%)		1 (14%)	
Chronic kidney disease		1 (5%)		1 (7%)		0 (0%)	
Solid tumor		5 (24%)		1 (7%)		4 (57%)	
Leukemia or lymphoma		1 (5%)		1 (7%)		0 (0%)	
Acquired immunodeficiency syndrome		0 (0%)		0 (0%)		0 (0%)	
Hypothyroidism		2 (10%)		1 (7%)		1 (14%)	
Connective tissue disease		3 (14%)		2 (14%)		1 (14%)	
Gastrointestinal disease		9 (43%)		7 (50%)		2 (29%)	
Genetic disease		1 (5%)		1 (7%)		0 (0%)	
Need for PN, n (%)	21		14		7		0.654
Ileus		0 (0%)		0 (0%)		0 (0%)	
Intolerance tube		1 (5%)		0 (0%)		1 (5%)	
Short bowel		8 (38%)		5 (36%)		3 (43%)	
Malabsorption		1 (5%)		1 (7%)		0 (0%)	
Malnutrition		0 (0%)		0 (0%)		0 (0%)	
Gastrointestinal Bleeding		0 (0%)		0 (0%)		0 (0%)	
Gastrointestinal obstruction		2 (19%)		2 (14%)		0 (0%)	
Dysmotility		5 (24%)		4 (28%)		1 (14%)	
Mucositis		0 (0%)		0 (0%)		0 (0%)	
Fistula		4 (19%)		2 (14%)		2 (29%)	
Chylothorax		0 (0%)		0 (0%)		0 (0%)	

Diarrhea		0 (0%)		0 (0%)		0 (0%)	
Anastomotic leak		0 (0%)		0 (0%)		0 (0%)	
Others (14)		0 (0%)		0 (0%)		0 (0%)	
Medications, n (%)	21		14		7		n/a
NSAID		8 (38%)		5 (36%)		3 (43%)	
Opioids		10 (48%)		6 (43%)		4 (57%)	
Antiemetics		8 (38%)		6 (43%)		2 (29%)	
Antidepressant		7 (33%)		4 (29%)		3 (43%)	
Antimotility		6 (29%)		6 (43%)		0 (0%)	
Blood glucose		2 (19%)		1 (7%)		1 (5%)	
Cardiovascular agents		8 (38%)		6 (43%)		2 (29%)	
Other gastrointestinal agents		16 (76%)		9 (64%)		7 (100%)	
Hormonals		3 (14%)		1 (7%)		2 (29%)	
Vitamins		17 (81%)		12 (86%)		5 (71%)	
Sleep		1 (5%)		1 (7%)		0 (0%)	
Anticonvulsants		3 (14%)		2 (14%)		1 (5%)	
Other		2 (19%)		0 (0%)		2 (29%)	

Figure S1: Frequency of ease of use for heparin lock

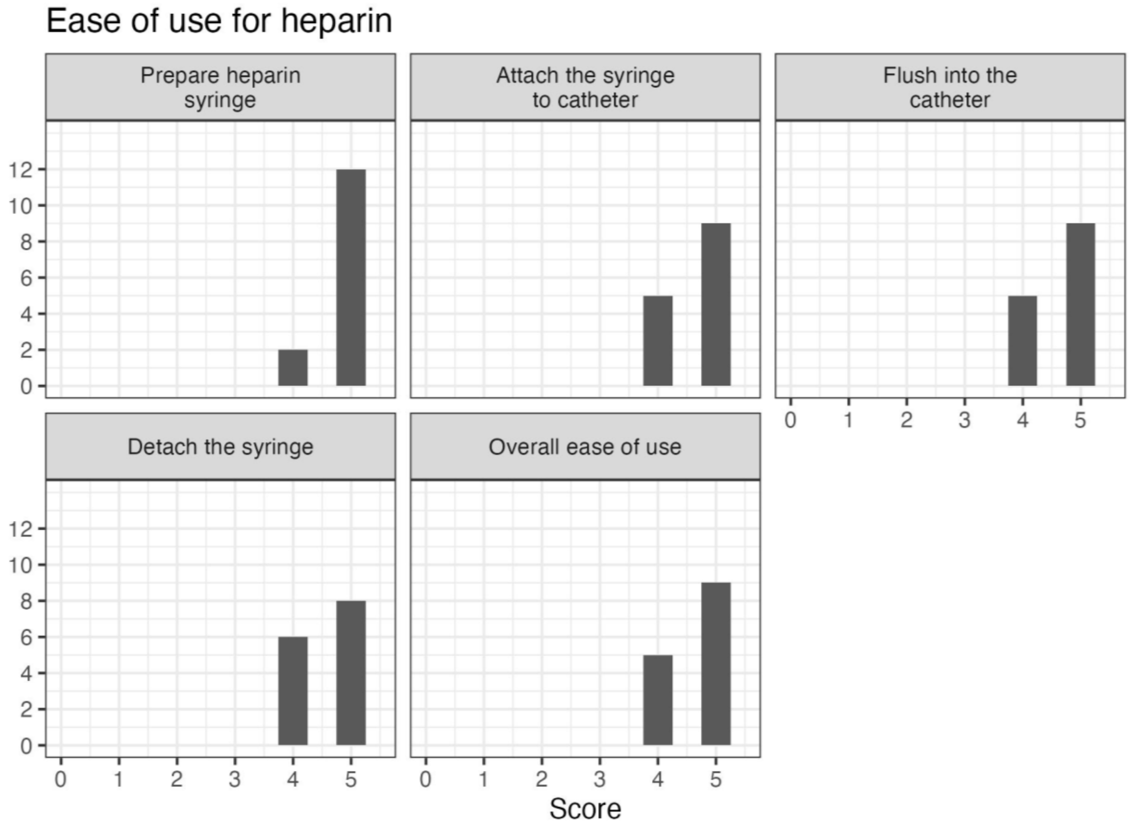

Questionnaire 1: End of Month 1

Regarding the preparation and use of the catheter lock solution, rate the ease of:

	Very hard 1	Hard 2	3	Easy 4	Very easy 5
Preparing the pre-filled heparin syringe					
Attaching the syringe to the catheter					
Flushing into the catheter					
Detaching the syringe					
Overall ease of use					

1. Rate your overall satisfaction when using this product:
   Unsatisfied Neutral Satisfied
   1 2 3
2. Rate the amount of time that it takes to use this product.
   Takes too long Doesn´t take long
   1 2 3
3. Would you continue to use this lock solution? Yes or No
   Why? Or why not?

Questionnaire 2: End of Month 2

Regarding the preparation and use of the catheter lock solution, rate the ease of:

	Very hard 1	Hard 2	3	Easy 4	Very easy 5
Opening the vial					
Attaching the syringe to the vial					
Withdrawing the solution into the syringe					
Detaching the syringe from the vial					
Injecting solution into the catheter (when disconnecting from PN)					
Overall ease of preparation					

1. Rate your overall satisfaction with preparing this product:
   Unsatisfied 1 — Neutral 2 — Satisfied 3
2. Rate the amount of time that it takes to prepare this product.
   Takes too long 1 — 2 — Doesn´t take long 3
3. Compared to your usual product (heparin), how easy is this product to prepare:
   Harder than heparin 1 — The same 2 — Easier than heparin 3
4. Would you continue to use this lock solution? Yes or No
   Why? Or why not?

Questionnaire 3: End of Month 3

Regarding the preparation and use of the catheter lock solution, rate the ease of:

	Very hard<br>1	Hard<br>2	3	Easy<br>4	Very easy<br>5
Opening the vial					
Attaching the syringe to the vial					
Flushing the solution through the catheter (before connecting the PN)					
Detaching the syringe from the vial					
Injecting solution into the catheter (when disconnecting from PN)					
Overall ease of preparation					

1. Rate your overall satisfaction with preparing this product:
   Unsatisfied Neutral Satisfied
   1 2 3
2. Rate the amount of time that it takes to prepare this product.
   Takes too long Doesn´t take long
   1 2 3
3. Compared to your usual product (heparin), how easy is this product to prepare:
   Harder than heparin The same Easier than heparin
   1 2 3
4. Would you continue to use this lock solution? Yes or No
   Why? Or why not?

Survey 1: End of Month 1

1. Record any new symptoms (pain, fever, etc.) on the day that they occur on the calendar before:

Monday	Tuesday	Wednesday	Thursday	Friday	Saturday	Sunday

2. Record the time it took to connect and disconnect the TPN on the first and last Monday of every month.
   Time to connect: Time to disconnect: Date:
   Time to connect: Time to disconnect: Date:
3. Has the patient hospitalized since the last phone call?

If yes, please specify the cause

4. Any line infection since the last phone call?
5. Any line thrombosis since last phone call?
6. Any new medication prescribed including herbal supplements?

If yes, please specify the medication group and name.

7. Any new antibiotic?

If yes, please specify the antibiotic name and duration.

8. Any change in vascular access?

If yes, please fill in the table below:

Reason:	
Type of vascular access	PICC<br>Tunneled catheter (Hickman)<br>Implanted catheter (Port-A-Cath)<br>Other
Location of catheter	
Number of lumens	

Survey 2: End of Month 2

1. Record any new symptoms (pain, fever, etc.) on the day that they occur on the calendar before:

Monday	Tuesday	Wednesday	Thursday	Friday	Saturday	Sunday

2. Record the time it took to connect and disconnect the TPN on the first and last Monday of every month.
   Time to connect: Time to disconnect: Date:
   Time to connect: Time to disconnect: Date:
3. Record the time it took to connect and disconnect the TPN on the first and last Monday of every month.
   Time to connect: Time to disconnect: Date:
   Time to connect: Time to disconnect: Date:
4. Has the patient hospitalized since the last phone call?

If yes, please specify the cause

5. Any line infection since the last phone call?
6. Any line thrombosis since last phone call?
7. Any new medication prescribed including herbal supplements?

If yes, please specify the medication group and name.

8. Any new antibiotic?

If yes, please specify the antibiotic name and duration.

9. Any change in vascular access?

If yes, please fill in the table below:

Reason:	
Type of vascular access	PICC<br>Tunneled catheter (Hickman)<br>Implanted catheter (Port-A-Cath)<br>Other
Location of catheter	
Number of lumens	

Survey 3: End of Month 3

1. Record any new symptoms (pain, fever, etc.) on the day that they occur on the calendar before:

Monday	Tuesday	Wednesday	Thursday	Friday	Saturday	Sunday

2. Record the time it took to connect and disconnect the TPN on the first and last Monday of every month.
   Time to connect: Time to disconnect: Date:
   Time to connect: Time to disconnect: Date:
3. Record the time it took to connect and disconnect the TPN on the first and last Monday of every month.
   Time to connect: Time to disconnect: Date:
   Time to connect: Time to disconnect: Date:
4. Has the patient hospitalized since the last phone call?

If yes, please specify the cause

5. Any line infection since the last phone call?
6. Any line thrombosis since last phone call?
7. Any new medication prescribed including herbal supplements?

If yes, please specify the medication group and name.

8. Any new antibiotic?

If yes, please specify the antibiotic name and duration.

9. Any change in vascular access?

If yes, please fill in the table below:

Reason:	
Type of vascular access	PICC<br>Tunneled catheter (Hickman)<br>Implanted catheter (Port-A-Cath)<br>Other
Location of catheter	
Number of lumens	
